# TD U-Net for Shell Segmentation and Thickness Evaluation in Core–Shell TiO_2_ TEM Images

**DOI:** 10.3390/ma18215007

**Published:** 2025-11-02

**Authors:** Zhen Ning, Chengjin Shi, Die Wu, Yu Zhang, Jiansu Pu, Yanlin Zhu

**Affiliations:** 1 Chengdu Advanced Metal Materials Industry Technology Research Institute Co., Ltd., Chengdu 610300, China; 2 State Key Laboratory of Vanadium and Titanium Resources Comprehensive Utilization, Chengdu 610031, China; 3 Pangang Group Research Institute Co., Ltd., Chengdu 610031, China; 4 School of Computer Science and Engineering, University of Electronic Science and Technology of China, Chengdu 611731, China

**Keywords:** titanium dioxide, transmission electron microscopy, deep learning, image segmentation, automated assessment

## Abstract

Titanium dioxide (TiO_2_) is widely used in coatings, plastics, rubber, papermaking, and other industries. The microstructural characteristics of its inorganic shell largely determine the overall performance of the product, significantly affecting optical behavior, dispersibility, weather resistance, and stability. Currently, coating quality evaluation in industry still relies primarily on manual inspection, lacking objective, standardized, and reproducible quantitative methods. This study focuses on lab-prepared core–shell TiO_2_ powders comprising a TiO_2_ core and a thin inorganic shell enriched in alumina/silica. This study presents Titanium Dioxide U-Net (TD U-Net)—a deep learning approach for transmission electron microscopy (TEM) image segmentation and shell thickness evaluation of core–shell structured TiO_2_ particles. TD U-Net employs an encoder–decoder architecture that effectively integrates multi-scale features, addressing challenges such as blurred boundaries and low contrast. We constructed a dataset of 1479 TEM images processed through a six-step workflow: image collection, data cleaning, annotation, mask generation, augmentation, and cropping. Results show that TD U-Net achieves a Dice coefficient of 0.967 for segmentation accuracy and controls shell-thickness measurement error within 5%, significantly outperforming existing image-processing models. An intelligent analysis system developed from this technology has been successfully applied to titanium dioxide product quality assessment, providing an efficient and reliable automated tool for coating-process optimization and quality control.

## 1. Introduction

Titanium dioxide (TiO_2_) is a white inorganic pigment featuring a high refractive index, excellent hiding power, and robust weather resistance. It is widely used across coatings, plastics, papermaking, rubber, cosmetics, food, and pharmaceuticals, and is an indispensable functional material in modern industry [1]. To further enhance performance, the industry commonly performs surface coating treatments on TiO_2_ particles to form inorganic shell layers (e.g., Al_2_O_3_, SiO_2_, ZrO_2_), thereby improving weather resistance, dispersibility, photocatalytic suppression, and compatibility with matrix materials [2]. In coatings and plastics in particular, the thickness, uniformity, and compactness of the shell layer directly influence gloss, hiding power, and long-term stability, and are important indicators of TiO_2_ quality grade [3].

In industrial practice, assessment of the density, uniformity, and thickness of the inorganic shell layer on TiO_2_ particles still relies mainly on qualitative manual judgments based on transmission electron microscopy (TEM) images [4]. Such assessments are highly subjective: outcomes can be influenced by the operator’s experience, background, and bias, leading to poor consistency and reproducibility. Additionally, precise quantitative analysis at the nanoscale is difficult—an issue that is especially limiting when fine control and optimization of particle coating quality are required in research and production. Moreover, manual judgments are inefficient; when processing large volumes of samples, they are time-consuming and prone to fatigue, further affecting accuracy and efficiency. Manual assessment, therefore, cannot meet the high-throughput and high-precision needs of modern materials science, constraining the pace and quality of research and product development.

Prior efforts to delineate microstructural regions in microscopy images span manual/threshold-based processing and classical machine-learning pipelines with hand-crafted features. More recently, convolutional neural networks (e.g., U-Net-style encoders/decoders) have been widely adopted for segmentation in materials microscopy, improving automation and accuracy; hybrid CNN–Transformer models further enhance global-context modeling for complex microstructures. These advances motivate our design while our work targets the specific challenges of shell delineation and thickness evaluation in TEM images of core–shell TiO_2_. A detailed discussion appears in Section 2 (Related Work). However, reliably delineating thin shells in TEM under blurred boundaries and low contrast remains challenging.

To address these issues, we develop an intelligent segmentation model for TiO_2_ TEM images that accurately extracts regions of interest—such as the TiO_2_ core and shell layer—thereby enabling precise, rapid, and automated support for process evaluation and quality control, and advancing the intelligence of materials design and production.

However, TEM images of coated particles typically exhibit blurred shell boundaries, low contrast, heterogeneous textures, and localization uncertainty, posing significant challenges for shell-layer segmentation and thickness evaluation. Traditional approaches to thickness measurement rely on manual calibration or basic image-processing operations. These methods are inherently subjective, limited in accuracy, and difficult to scale for fast, precise industrial evaluation.

In recent years, convolutional neural networks (CNNs) have excelled in image-segmentation tasks. CNN-based methods are adept at local modeling and can identify microstructural features such as grains, phase boundaries, and pores in microscopy images, enabling efficient extraction and automated analysis of complex image information [5]. Yet fixed convolutional kernels restrict the acquisition of long-range dependencies and limit global modeling capacity. Attention mechanisms, dilated convolutions, and spatial pyramid techniques alleviate—but do not fully solve—this problem. Transformers, leveraging self-attention [6], emphasize global context and long-range dependencies, improving contextual modeling; nevertheless, they are relatively insensitive to local positions and can under-represent local details.

Against this backdrop, we propose TD U-Net—an automated segmentation and thickness-evaluation method for inorganic shells on TiO_2_ particles that integrates deep learning with quantitative image analysis. Our main contributions are: (1) a high-quality TEM dataset covering diverse shell states with expert annotations and standardized preprocessing; (2) a dual-path TD U-Net architecture that introduces multi-scale feature extraction and skip connections to strengthen global modeling while preserving local details for precise shell-region segmentation; (3) a thickness-regression and density-estimation module for automated statistics of shell thickness, uniformity, and compactness; and (4) an end-to-end automated software system that takes TEM images as input and outputs shell-thickness evaluations, validated on real TiO_2_ product images to markedly improve efficiency and consistency.

## 2. Related Work

### 2.1. Traditional Machine-Learning Models for Microstructure Recognition

Microstructure recognition in materials imaging has historically relied on manual identification, traditional image processing, and machine-learning-based automation. Manual recognition suffers from subjectivity, difficulties in precise quantification, and inefficiency. Traditional image processing—e.g., edge detection and thresholding—can automate certain cases, but typically leverages a single image feature and lacks robustness for complex backgrounds.

Classical machine-learning methods such as support-vector machines (SVMs) and random forests have been widely applied to the segmentation of material microstructures. Bulgarevich et al. [7] used random forests for reliable automated segmentation of metallurgical microstructures, achieving area fractions and locations that closely matched manual inspection, thus demonstrating utility for metals. In SEM image analysis, decision-tree-based methods like random forests (RFs) and gradient boosting machines (GBMs), coupled with texture features such as gray-level co-occurrence matrices (GLCMs), effectively distinguish martensite, upper bainite, and lower bainite in steels [8].

These approaches typically extract hand-crafted features (e.g., texture or morphology) and feed them into a classifier or regressor. For instance, combining GLCM and LBP features with an SVM has successfully recognized several bainite types (granular, degenerated upper bainite, lower bainite) [8]. However, when facing high-dimensional and highly complex image data, hand-crafted-feature pipelines often struggle with efficiency and generalization.

Moreover, SVMs and random forests exhibit different strengths: RFs often excel in multi-class problems and can handle mixed numerical and categorical features, while SVMs are strong in high-dimensional or sparse settings (e.g., document classification). RFs are typically faster than nonlinear SVMs on datasets exceeding ~10,000 samples.

### 2.2. Deep Learning for Microstructure Recognition

Deep learning has opened new avenues for microstructure recognition [9,10]. CNNs, with strong local modeling capacity, are widely used for segmentation. In materials science, U-Net [11]—with its symmetric encoder–decoder and skip connections that mitigate detail loss from down-sampling—has become a de facto standard for microstructure segmentation.

DeCost et al. [12] applied PixelNet to segment constituents in ultrahigh-carbon-steel micrographs. By building a “hypercolumn” for each pixel from features across layers, PixelNet classifies pixels using standard classifiers, showing advantages on complex steel microstructures. Azimi et al. [13] integrated fully convolutional networks to segment martensite, tempered martensite, bainite, and pearlite in steel SEM images, achieving ~94% accuracy and significantly improving automated phase segmentation.

U-Net variants continue to advance performance: U-Net++ [14] uses dense skip connections to reduce semantic gaps between encoder and decoder, improving segmentation of subtle structures and blurred boundaries. Still, CNN-based methods lack global context modeling. Researchers have explored attention, dilations, and spatial pyramids to enhance context awareness, but challenges remain for complex, low-contrast TEM images of coated TiO_2_.

Transfer learning is also widely used. Unsupervised domain adaptation can outperform simple fine-tuning for classification and segmentation in materials datasets [15]. Highest gains arise when the source domain is visually similar to the target (e.g., other microscopy images rather than ImageNet).

### 2.3. Transformers for Microstructure Segmentation

Transformers [6], introduced from NLP to vision, capture global context via self-attention and address CNNs’ limitations in global modeling. TransUNet [16] first fused CNN features with Transformer encoders, combining local detail sensitivity with long-range dependency modeling. Pretraining encoders on large microscopy datasets further boosts segmentation for microstructures compared with pretraining on natural images [17], highlighting the value of domain-specific pretraining.

Hybrid CNN-Transformer models (e.g., Swin-UNet [18]) replace convolutional blocks with Swin Transformer blocks while retaining the U-shaped topology, improving capability on complex microstructures. For complex steel microstructures, Transformer self-attention better captures spatial relations among phases [19].

Despite progress, Transformer-based models can be computationally heavy and sometimes under-resolve fine textures and low-contrast areas in TEM. Axially enhanced Transformers—performing attention along height and width—can improve pixel-level modeling and boundary accuracy [20]. Human-in-the-loop frameworks that combine weak supervision and active learning also reduce annotation cost while achieving precise segmentation [21]. These insights motivate our TD U-Net design, which fuses CNN-style local detail modeling and Transformer-style global context while accounting for the specific challenges of TEM imaging (low contrast, noise, blurred boundaries) Similar conclusions on microstructure recognition by deep learning were also reported in [22].

## 3. Methods

### 3.1. Samples

The materials analyzed are lab-prepared prototype core–shell TiO_2_ powders used for method development. The core is TiO_2_ (rutile-based), and the shell is a thin inorganic layer enriched in alumina and/or silica at the particle rim. These laboratory-scale samples were supplied for algorithm development; specific downstream application scenarios and detailed synthesis routes are outside the scope of this study.

### 3.2. TEM Sample Preparation and Imaging

Powders were placed in centrifuge tubes and dispersed in absolute ethanol (typical working concentration: 0.1–0.5 mg mL^−1^). The suspensions were bath-sonicated for 30 min, and 3–5 μL of the supernatant was drop-cast onto carbon-coated copper TEM grids (e.g., 300-mesh). The grids were air-dried at room temperature prior to imaging. Bright-field TEM images were acquired at an accelerating voltage of 120 kV, with a nominal resolution of ~0.24 nm and a magnification range of ~6 × 10^4^ to 1 × 10^5^. All chemicals and consumables were purchased from commercial suppliers (Chengdu, China) and used as received.

### 3.3. Workflow and Instrumentation

**Figure 1:**
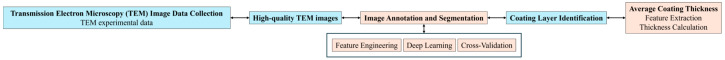
Workflow of this study.

Figure 1 outlines the complete workflow of our method, from data collection to thickness evaluation. The workflow consists of (1) TEM image acquisition, where high-resolution experimental data are collected; (2) image quality control, in which low-contrast or noisy images are filtered to ensure data reliability; (3) manual annotation and segmentation, where shell layers are labeled to generate training masks; (4) deep learning model training and inference, using feature engineering, cross-validation, and optimization strategies; (5) shell layer identification, where the trained model performs automated segmentation on new samples; and (6) thickness evaluation, involving skeleton-based morphological analysis and statistical quantification (e.g., mean thickness, standard deviation, coefficient of variation).

**Figure 2:**
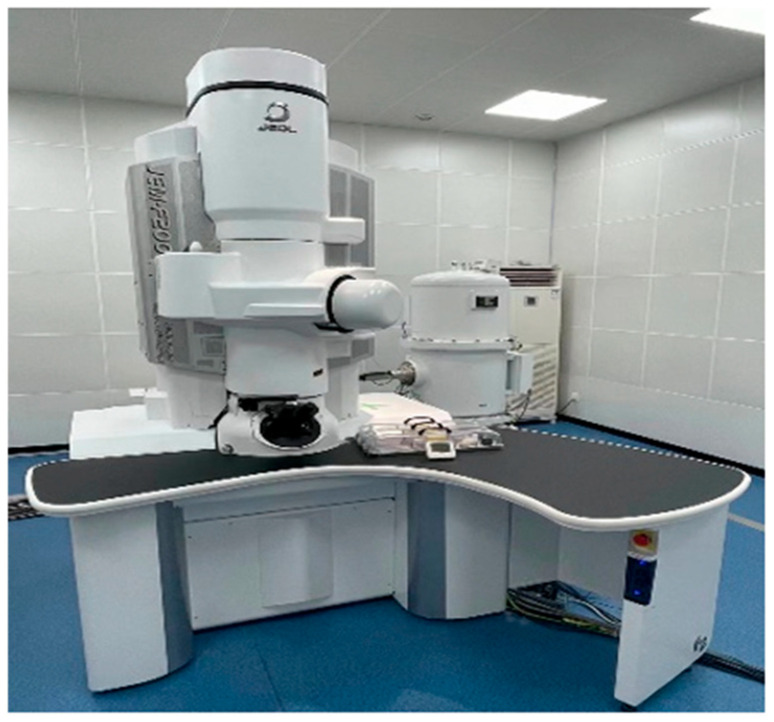
JEM-F200 field emission transmission electron microscope (FE-TEM).

TEM images were acquired using a JEM-F200 field-emission transmission electron microscope (JEOL, Tokyo, Japan), as shown in Figure 2.

Prior to annotation and training, raw TEM images were screened according to predefined inclusion/exclusion criteria. Images were retained only if all of the following were satisfied: (i) a resolvable shell–core boundary under the stated acquisition settings; (ii) global focus, defined as ≥ 80% of the field in acceptable focus and free of drift/motion blur; and (iii) absence of severe artifacts such as heavy contamination, charging, or saturation. Images failing any criterion were excluded after reviewer inspection.

### 3.4. Model Architecture

**Figure 3:**
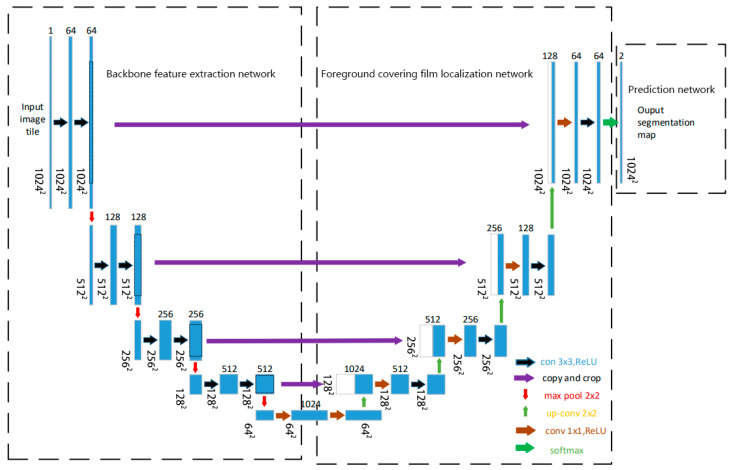
Network architecture of this study.

We design TD U-Net to address the challenges of low contrast, blurred boundaries, and fine-scale structural variability in TEM images of core–shell TiO_2_ particles. The model is structured into three functionally distinct components: (1) a backbone feature extraction network that captures multi-scale representations from the input image, (2) a segmentation path focusing on shell boundary delineation that emphasizes boundary-sensitive feature mapping, and (3) a prediction head that produces shell segmentation masks. The overall architecture adopts a U-Net-like encoder–decoder topology with skip connections and feature fusion mechanisms. Figure 3 shows the complete network layout. This architecture is tailored to cope with the low contrast, blurred boundaries, and irregular morphologies often observed in TEM images of core–shell nanoparticles.

### 3.5. Network Design

TD U-Net employs a symmetric encoder–decoder architecture with skip connections, enabling the integration of low-level spatial details and high-level semantic features. This design is well-suited to preserve fine boundary information while maintaining global context awareness.

The network takes a single-channel 1024 × 1024 TEM image as input. The encoder path (contracting path) consists of four convolutional blocks:

Block 1: two 3 × 3 convolutions with 64 channels, followed by 2 × 2 max-pooling, producing a feature map of size 512 × 512 × 64;

Block 2: two 3 × 3 convolutions with 128 channels, followed by max-pooling, resulting in 256 × 256 × 128;

Block 3: two 3 × 3 convolutions with 256 channels, downsampled to 128 × 128 × 256;

Block 4: two 3 × 3 convolutions with 512 channels, followed by pooling to 64 × 64 × 512.

The bottleneck layer applies two 1 × 1 convolutions with 1024 channels at the 64 × 64 resolution.

The decoder path (expanding path) performs upsampling and feature fusion through skip connections:

Block 1: the 64 × 64 × 1024 bottleneck output is upsampled to 128 × 128 × 1024, fused with encoder features via a 1 × 1 convolution, followed by a 3 × 3 convolution with 512 channels;

Block 2: upsampling to 256 × 256 × 512, followed by two 1 × 1 fusions and a 3 × 3 convolution with 256 channels;

Block 3: upsampling to 512 × 512 × 256, concatenated with encoder features (128 channels), fused via 1 × 1 convolution and refined by a 3 × 3 convolution with 128 channels;

Block 4: upsampling to 1024 × 1024 × 128, concatenated with encoder features (64 channels), followed by 1 × 1 fusion and a 3 × 3 convolution with 64 channels.

Finally, a 1 × 1 output layer maps the 64-channel feature map to two output channels (foreground and background), followed by a softmax activation to produce the segmentation map.

### 3.6. Thickness-Evaluation Module

**Figure 4:**
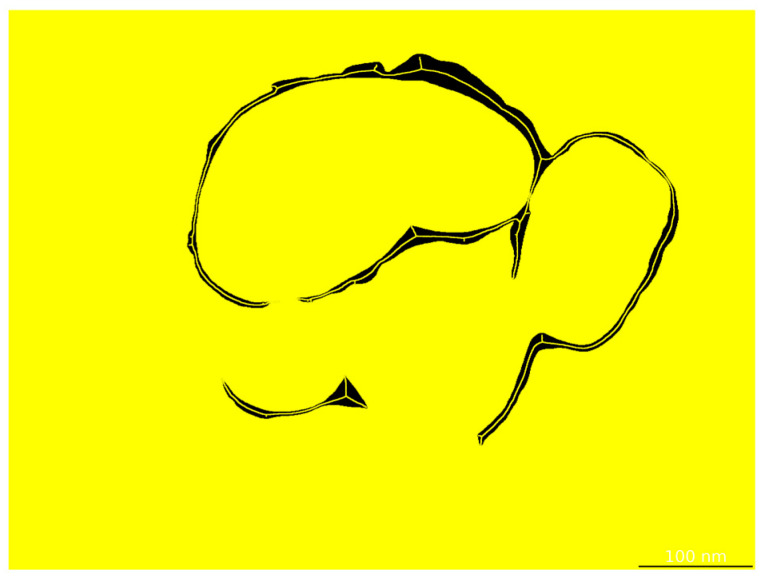
Skeletonization of the shell layer by the thickness evaluation module.

After segmentation, shell thickness is quantified via skeleton-based morphological analysis. The centerline of the segmented shell region is extracted (Figure 4), and the local thickness at each skeleton point is computed as twice the shortest distance to the boundary. A full-thickness distribution is obtained, and key statistics—including mean, standard deviation, and coefficient of variation—are reported to evaluate shell uniformity.

### 3.7. Data Augmentation

To improve generalization and robustness under limited annotated data, we apply a series of data augmentation strategies tailored to the characteristics of TEM imaging and nanoparticle morphology. These include the following: (i) elastic deformations to simulate shape variability introduced during sample preparation; (ii) geometric transformations such as random rotations (0–360°), horizontal and vertical flips, and scaling (0.8–1.2×), to encourage spatial invariance; (iii) photometric perturbations, including random adjustments of brightness and contrast (±20%) and gamma correction (0.8–1.2), to emulate imaging condition variations; and (iv) noise injection, using Gaussian noise and salt-and-pepper noise, to enhance model robustness against acquisition noise.

### 3.8. Automatic Scale-Bar Recognition

**Figure 5:**
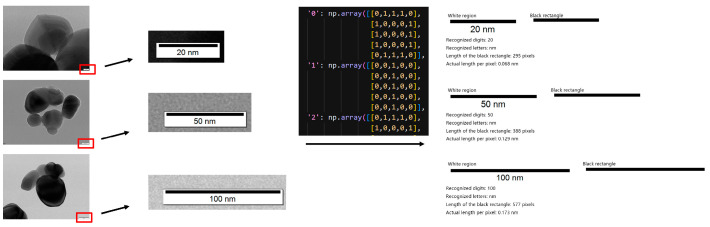
Automatic scale bar recognition module and its working principle.

To convert pixel distances into physical thickness values (in nm), TD U-Net incorporates an automatic scale-bar recognition module. Image-processing routines first localize the scale-bar region; OCR then parses its text to establish the pixel-to-physical mapping. The system robustly recognizes common scale bars such as 20 nm, 50 nm, and 100 nm (Figure 5). This module enables accurate and automated unit calibration, eliminating the need for manual measurements.

## 4. Experiments and Results

### 4.1. Dataset Construction

**Figure 6:**
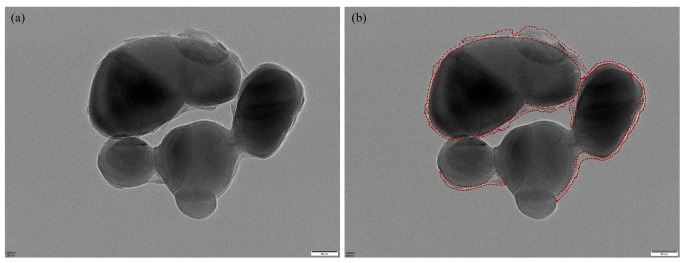
Manual annotation example: (**a**) original image; (**b**) Labelme annotation, where red lines indicate the annotated shell boundaries.

We constructed a standardized TEM image dataset comprising representative core–shell TiO_2_ samples collected from a vanadium–titanium magnetite steel plant. The original grayscale images were acquired at magnifications of 60,000×, 80,000×, or 100,000×, with dimensions of 5120 × 3840 pixels. Regions of interest—primarily shell layers—were manually annotated by experienced researchers using the Labelme tool, as illustrated in Figure 6, which provides a representative example of the manual annotation process. Annotation was particularly challenging due to complex particle morphologies, overlapping structures, and low-contrast regions common in TEM imaging.

**Figure 7:**
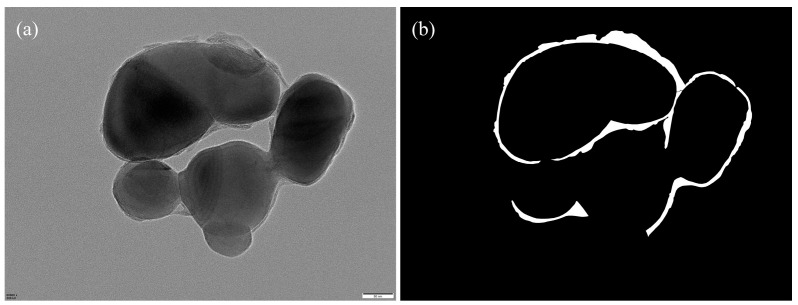
(**a**) Original image; (**b**) corresponding binary segmentation mask.

**Figure 8:**
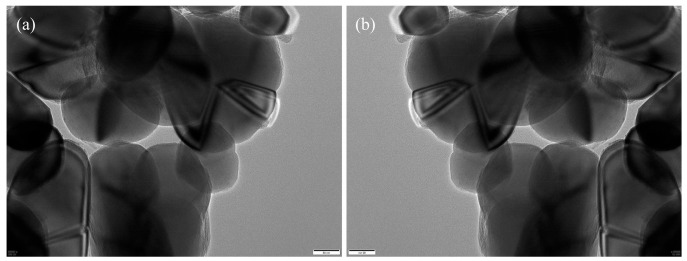
Data augmentation examples: (**a**) original; (**b**) augmented image.

The annotated regions were programmatically converted into binary segmentation masks via Python (v.3.12) scripts (Figure 7), forming the ground truth for model training. To increase the variability and improve the model’s robustness against morphological and imaging variations, we applied a series of data augmentation strategies—including geometric transformations, photometric perturbations, and noise injection—on the annotated images (Figure 8).

**Figure 9:**
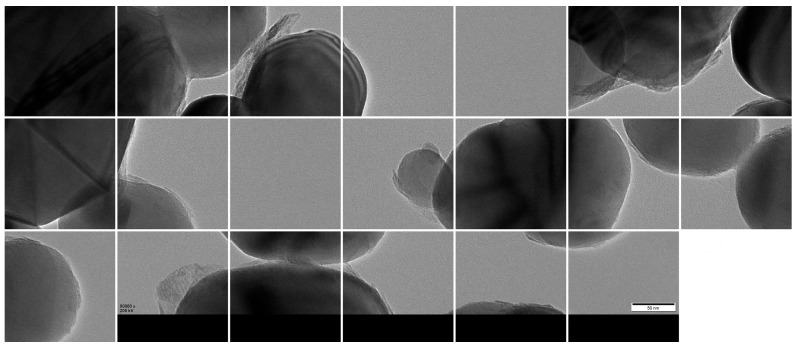
Cropped 1024 × 1024 patches from original TEM images.

Given GPU memory constraints, the high-resolution images were further partitioned into 1024 × 1024 patches using a sliding window approach with overlap, resulting in 3000 valid sub-images (Figure 9). The dataset was then randomly divided into training, validation, and test sets using a 7:2:1 ratio, yielding 2100, 600, and 300 patches, respectively.

Additionally, 14 large-format images (5120 × 3840) were reserved as representative test samples to qualitatively assess model performance in practical scenarios.

### 4.2. Experimental Setup

TD U-Net was implemented in PyTorch (v2.5.1) and trained on two NVIDIA A100 GPUs (80 GB VRAM each). Training was performed for 200 epochs with a mini-batch size of 16 using the Adam optimizer (initial learning rate 1 × 10^−3^, with scheduled decay). A hybrid loss function combining cross-entropy and Dice loss was employed to improve convergence and address class imbalance.

### 4.3. Evaluation Metrics

To quantitatively evaluate segmentation performance, we adopted two widely used metrics: the Dice similarity coefficient (*DSC*) and the intersection-over-union (*IoU*). These metrics are particularly suitable for binary segmentation tasks with class imbalance and small foreground regions, as is typical in TEM imaging of core–shell nanoparticles.

The *IoU* evaluates the degree of overlap between the predicted segmentation mask and the ground-truth annotation. It is defined as the ratio of the intersection to the union of the predicted region *P* and the ground-truth region *G*, as follows:

(1) $$IoU=\frac{\left|P\cap G\right|}{\left|P\cup G\right|}$$

The Dice similarity coefficient, also known as the F1 score in binary segmentation, provides another measure of region overlap. It represents the harmonic mean of precision and recall, and is defined as:

(2) $$DSC=\frac{2\left|P\cap G\right|}{\left|P\right|+\left|G\right|}$$

Both *IoU* and *DSC* range from 0 to 1, with higher values indicating better segmentation quality. In our experiments, we report both metrics to comprehensively evaluate segmentation accuracy and robustness.

### 4.4. Segmentation Performance

**Figure 10:**
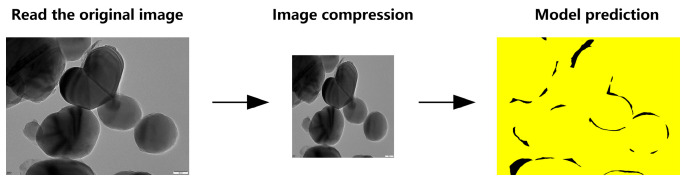
Schematic workflow of TD U-Net (Method 1).

To handle full-resolution TEM images (5120 × 3840 pixels), we evaluated two inference strategies based on the TD U-Net model. Method 1 directly downsamples the original image to 1024 × 1024 resolution, performs segmentation, and then upscales the predicted mask back to its original size (Figure 10).

While this approach achieves relatively high accuracy on most of the 14 test samples (with Dice coefficients generally above 0.85 and *IoU* scores mostly exceeding 0.75), its performance degrades on certain challenging cases—particularly samples 8 and 10—likely due to complex morphological features or low contrast.

**Figure 11:**
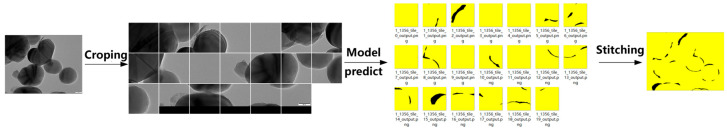
Schematic workflow of TD U-Net (Method 2).

Method 2, in contrast, divides each high-resolution image into multiple 1024 × 1024 patches using a sliding window, applies segmentation to each patch, and reconstructs the full-size mask by stitching the predicted segments (Figure 11).

**Figure 12:**
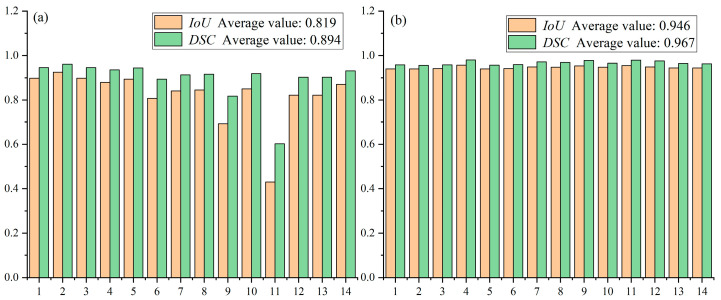
Performance comparison between method 1 and method 2 on the test set: (**a**) average performance of Method 1; (**b**) average performance of Method 2.

This strategy yields more consistent performance across the dataset, with Dice scores ranging from approximately 0.88 to 0.95 and *IoU* scores from 0.80 to 0.90 (Figure 12). The superior stability of Method 2 can be attributed to its ability to preserve fine details that are otherwise lost during downsampling in Method 1. These results demonstrate that the TD U-Net architecture effectively captures both local details and global contextual information, and highlight the benefits of the proposed feature-fusion strategy.

To further benchmark segmentation performance, we compared our model against two additional baselines: a traditional image processing method and a fine-tuned Segment Anything Model (SAM). Although SAM is a powerful foundation model, its performance was limited when applied to the low-contrast and highly detailed TEM images in our dataset. As summarized in Table 1, TD U-Net (Method 2) outperformed all other approaches, achieving the highest Dice (0.967) and *IoU* (0.946) scores. This confirms the effectiveness of the proposed intelligent segmentation and reconstruction framework.

### 4.5. Thickness Evaluation Results

Following the segmentation stage, shell thickness was quantitatively evaluated using the skeleton-based morphological analysis described in Section 3.6. The segmented shell region is thinned to extract a centerline (“fishbone” skeleton), and the local thickness at each point along this centerline is computed as twice the shortest Euclidean distance to the shell boundary. Pixel-level measurements are then converted into nanometers using the scale-bar recognition module.

**Figure 13:**
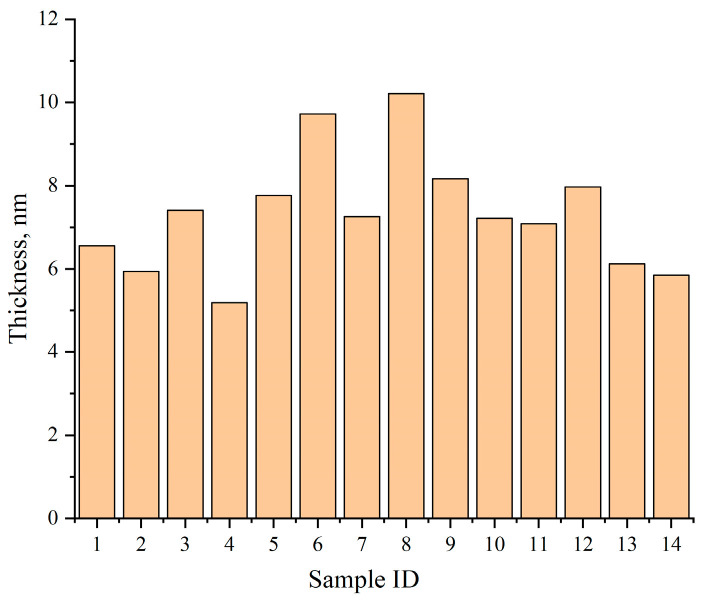
Average shell thickness across 14 representative test samples as computed by TD U-Net.

Figure 13 summarizes the average shell thickness values across 14 held-out test samples, with results ranging from 5.18 nm to 10.21 nm. Compared with manual measurements, the deviation of the automated results remains within 5%, validating the method’s accuracy.

In addition to the mean thickness, the system also reports the standard deviation, minimum, maximum, and coefficient of variation (CV = std/mean) for each image and region. The CV serves as a robust quantitative indicator of shell uniformity and aligns well with expert assessments, making the system suitable for large-scale quality screening and comparative analysis.

### 4.6. Demonstration on Industrial Samples

To demonstrate the practical utility of our method, we deployed the proposed TD U-Net–based pipeline as a standalone software module and applied it to assess the shell quality of a commercial TiO_2_ product (CR350) produced at a vanadium–titanium magnetite steel plant.

**Figure 14:**
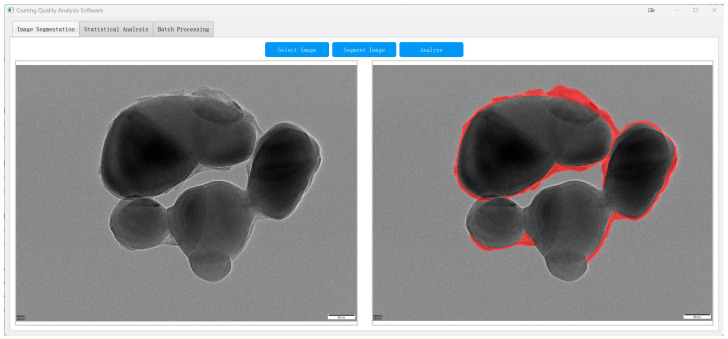
TD U-Net segmentation of an industrial TEM image ((**left**): original; (**right**): overlay of the predicted shell parts (red)).

As shown in Figure 14, the system enables automatic segmentation of core–shell TiO_2_ particles in raw TEM images, with shell boundaries delineated in red. This facilitates subsequent quantitative analysis across the entire field of view.

**Figure 15:**
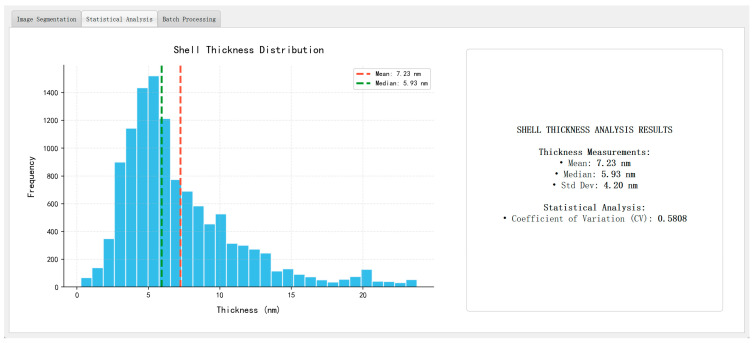
Automated analysis outputs ((**left**): particle size histogram; (**right**): summary statistics panel).

Figure 15 presents representative analysis outputs, including a log-normal particle size distribution (dominant range: 20–40 nm), a pseudo-color map of shell thickness, and a summary panel reporting key morphological statistics such as mean particle size, average shell thickness, standard deviation, and coefficient of variation (CV). The thickness values are computed using the skeleton-based method introduced in Section 3.6, with physical unit conversion achieved via the scale-bar recognition module described in Section 3.8.

**Figure 16:**
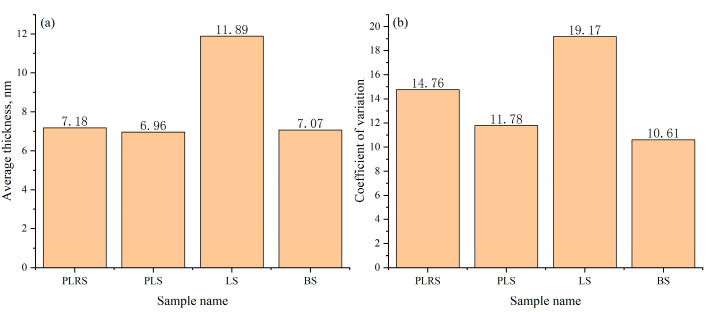
Comparison of average shell thickness (**a**) and Coefficient of variation (**b**) across four sample types. This figure assesses robustness/QA; no physico-chemical comparison is intended or implied.

To evaluate cross-sample robustness and quality-control utility, we analyzed four independent TEM datasets obtained from different sources: production-line reference sample (PLRS), production-line sample (PLS), laboratory sample (LS), and benchmark sample (BS) (Figure 16). These datasets were compiled to test generalizability across real-world acquisition provenance (in-house and market-purchased). The analysis focuses on image-derived shell-thickness statistics rather than physico-chemical differences among materials. The LS group exhibited the highest mean thickness (11.89 nm), whereas PLRS, PLS, and BS were relatively consistent near 7 nm (7.18, 6.96, and 7.07 nm, respectively). To characterize within-dataset uniformity, we report the coefficient of variation (CV = std/mean): PLRS 14.76%, PLS 11.78%, LS 19.17%, and BS 10.61%. Thus, despite its larger mean thickness, the LS dataset shows the largest intra-dataset variability (highest CV), while the BS dataset shows the lowest CV. These results indicate that the system captures both level (mean) and uniformity (CV) differences across datasets and can support retrospective QA and screening across heterogeneous TEM inputs. All evaluations were performed offline on pre-acquired high-resolution TEM images without modifying the pipeline, underscoring generalizability.

## 5. Conclusions

We proposed TD U-Net, a deep-learning-based method for automated segmentation and shell-thickness evaluation of core–shell TiO_2_ particles in high-resolution TEM images. The key innovations include the following:

(i) A dual-path architecture with multi-scale feature fusion and attention, which significantly enhances fine-boundary recognition while balancing accuracy and efficiency;

(ii) A skeleton- and distance-transform–based thickness quantification module that enables pixel-level shell thickness analysis and statistical evaluation;

(iii) An integrated software system supporting one-click processing, visualization, and reporting for large-scale TEM datasets.

The method was validated on a curated dataset and tested on real-world industrial samples. All industrial evaluations were performed offline using pre-acquired TEM images, without modifying the system pipeline. The results demonstrate that the system can generalize well across varied samples and offers a reliable, automated alternative to manual inspection.

Future work includes improving accuracy for ultra-thin shells, exploring 3D reconstruction based on 2D segmentation, integrating multimodal data (e.g., SEM, XRD), and embedding the shell-thickness evaluation pipeline into online monitoring systems for real-time process optimization.

## Figures and Tables

**Table 1:** Comparison of segmentation performance across different methods on the test set.

Method	*DSC*	*IoU*
Traditional method	0.787	0.654
SAM model	0.853	0.748
TD U-Net (Method 1)	0.894	0.819
**TD U-Net (Method 2)**	**0.967**	**0.946**

## Data Availability

The original contributions presented in this study are included in the article. Further inquiries can be directed to the corresponding author.
